# In Vivo Characterization of Magnetic Inclusions in the Subcortex From Nonexponential Transverse Relaxation Decay

**DOI:** 10.1002/nbm.70051

**Published:** 2025-05-05

**Authors:** Rita Oliveira, Quentin Raynaud, Ileana Jelescu, Valerij G. Kiselev, Evgeniya Kirilina, Antoine Lutti

**Affiliations:** ^1^ Laboratory for Research in Neuroimaging, Department of Clinical Neuroscience Lausanne University Hospital and University of Lausanne Lausanne Switzerland; ^2^ Department of Radiology Lausanne University Hospital (CHUV) and University of Lausanne Lausanne Switzerland; ^3^ Division of Medical Physics, Department of Radiology, Faculty of Medicine University of Freiburg Freiburg Germany; ^4^ Department of Neurophysics Max Planck Institute for Human Cognitive and Brain Sciences Leipzig Germany

**Keywords:** brain iron, dopaminergic neurons, nonexponential decay, quantitative MRI, $R_{2}^{*}$, relaxometry, substantia nigra, transverse relaxation

## Abstract

According to theoretical studies, MRI signal decay due to transverse relaxation in brain tissue with magnetic inclusions (e.g., blood vessels and iron‐rich cells) is expected to follow a transition from Gaussian behaviour at short echo times to exponential behaviour at longer times. The decay parameters carry information about the inclusions (e.g., size and volume fraction) and provide unique insights into brain microstructure. However, gradient‐echo decays typically only capture the long‐time exponential behaviour. We provide experimental evidence of nonexponential transverse relaxation decay in human subcortical grey matter from in vivo MRI data acquired at 3 T, allowing the subsequent characterization of the magnetic inclusions. Gradient‐echo data were collected with short interecho spacings, minimal echo time (1.25 ms) and novel acquisition strategies to mitigate motion and cardiac‐induced effects. The data were fitted using exponential and nonexponential models that describe the impact of magnetic inclusions on the MRI signal. Nonexponential models provided superior fits. The strongest deviations from exponential were detected in the substantia nigra and globus pallidus. Numerical simulations of the signal decay from histological maps of iron concentration in the substantia nigra replicated the experimental data, highlighting that non‐haem iron can be at the source of the nonexponential decay. To investigate the potential of nonexponential decays to characterize brain microstructure, we estimated the properties of the underlying inclusions using two analytical models. Under the static dephasing regime, the magnetic susceptibility and volume fractions of the inclusions ranged between 1.8–4 and 0.02–0.04 ppm, respectively. Alternatively, under the diffusion narrowing regime, the typical inclusion size was ~2.4 μm. Both simulations and experimental data suggest an intermediate regime with a non‐negligible effect of water diffusion. Nonexponential transverse relaxation decay allows to characterize the spatial distribution of magnetic material within subcortical tissue with increased specificity, with potential applications for Parkinson's disease and other pathologies.

## Graphical abstract

Nonexponential transverse relaxation decay allows to characterize the spatial distribution of magnetic material within subcortical grey matter tissue.

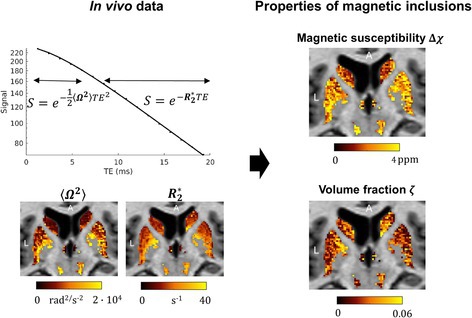

**Abbreviations**
AIC: Akaike information criterion
DNR: diffusion narrowing regime
GP: globus pallidus
MSE: mean squared error
PIXE: proton‐induced X‐ray emission microscopy
SDR: static dephasing regime
SN: substantia nigra

## 1 Introduction

The decay of gradient‐echo magnetic resonance imaging (MRI) data due to transverse relaxation is widely considered to follow an exponential behaviour with a rate $R_{2}^{*}$. Estimates of $R_{2}^{*}$ correlate with iron concentration within brain tissue [1, 2, 3, 4], a property of primary importance for the study of the brain (see [5, 6] for a review). Iron plays a crucial role in various biological processes such as myelin synthesis, energy production, neurotransmitter synthesis and signalling [7, 8]. Therefore, some cell types including oligodendrocytes, microglia and dopaminergic neurons exhibit elevated cellular iron concentrations. Abnormal accumulation of iron constitutes a hallmark of neurodegenerative disorders such as Parkinson's disease [9, 10, 11] and can be monitored non‐invasively in patients using $R_{2}^{*}$ mapping data [12, 13]. Empirical models have been proposed to link $R_{2}^{*}$ to the overall iron concentration [14, 15, 16]. However, these models lack a biophysical foundation crucial for specificity and fail to capture the characteristics of iron‐rich cells in the tissue.

Microscopic inclusions of magnetic material within brain tissue, such as iron‐rich cells, myelin or blood vessels, induce microscopic inhomogeneities of the magnetic field, which can result in a nonexponential gradient‐echo signal decay [5, 17, 18, 19, 20, 21, 22, 23, 24, 25, 26]. According to theoretical studies, signal decays that result from these magnetic field inhomogeneities display a Gaussian behaviour at short echo times followed by an exponential behaviour at longer echo times [17, 18, 22, 24, 25]. Combined, the coefficients that describe the Gaussian and exponential behaviours carry complementary information about those inclusions (e.g., volume fraction, magnetic susceptibility and size). If measured experimentally, these coefficients allow the assessment of the inclusions with improved specificity, offering valuable insights into the cellular underpinnings of neurodegenerative diseases. However, although this nonexponential behaviour has been observed in phantoms containing cylindrical fibres [27], suspensions of paramagnetic beads [28], ex vivo brain samples [22] and in vivo blood vessels [29], no such evidence exists in iron‐rich subcortical grey matter.

The characterization of the magnetic inclusions at the source of nonexponential signal decay requires biophysical models of transverse relaxation that establish a quantitative link between the measured MRI data and the properties of the inclusions within brain tissue (e.g., volume fraction and magnetic susceptibility). These models capture two phenomena. One is the distribution of Larmor frequencies experienced by water molecules due to the microscopic spatial magnetic field inhomogeneities induced by the inclusions. These inhomogeneities are static, and their effect on the MRI signal is in principle refocusable using spin echoes. The other is the temporal effects of water diffusion across this inhomogeneous field, which are non‐refocusable. Both spatial and temporal effects contribute to signal decay (see [17] for a review). However, analytical expressions of the MRI signal may only be derived from these biophysical models under two mutually exclusive limiting cases. In one case (static dephasing regime [SDR]), the spatial inhomogeneities constitute the dominant mechanism underlying signal decay [18, 30]. In the other (diffusion narrowing regime [DNR]), the temporal effects dominate [19, 22, 24, 25, 31].

The question of which regime is more suitable to describe the biophysics of transverse relaxation within brain tissue is a topic of debate and depends on the magnetic field strength and brain region under consideration [32, 33, 34]. In the subcortex, quantitative assessment of iron distribution at the microscopic scale revealed a complex distribution of paramagnetic iron, characterized by a substantial amount dispersed diffusely throughout the tissue, in addition to localized iron‐rich cells [35, 36, 37]. Biophysical models informed by such detailed quantitative measurements have demonstrated that the SDR is suitable to describe the contribution of dopaminergic neurons in the substantia nigra (SN) at field strengths of 7 T or above [33]. At lower field strengths, diffusion needs to be taken into account [37]. Because such detailed findings have not been presented for other subcortical nuclei and populations of iron‐rich cells, our understanding of which relaxation regime dominates remains fragmented.

In this work, we provide experimental evidence of nonexponential MRI signal decay due to transverse relaxation in subcortical brain regions in gradient‐echo data acquired in vivo at 3 T. We fitted the signal decay with an empirical expression and with theoretical models of the effect of magnetic inclusions on the MRI signal [22, 25, 31]. From the value of the Gaussian and exponential parameters of the signal decay, we estimated the properties of the magnetic inclusions under the assumption of the SDR and DNR. For the SN, these estimates were compared with the microscopic distribution of iron known from analyses of ex vivo brain tissue.

## 2 Theory

### 2.1 Transverse Relaxation in the Presence of Magnetic Inclusions

Differences between existing biophysical models of the effect of magnetic inclusions on transverse relaxation mainly involve the dominating dephasing regime (SDR or DNR) and secondary assumptions about the spatial distribution of magnetic material at the microscopic scale. In the DNR, the models that describe the effect of weak magnetic field inhomogeneities on the MRI signal differ in the form of the autocorrelation function of the Larmor frequency experienced by diffusing spins over time. In the model of Anderson and Weiss [31] (AW), this autocorrelation function is assumed to take an exponential form. In the model of Sukstanskii and Yablonskiy [25] and Jensen and Chandra [22] (JC), the autocorrelation function was derived analytically from Gaussian water diffusion within the tissue. All existing models predict asymptotic behaviours of the signal decay of the following forms:

(1) $$S\approx S_{0}\exp\left(-\frac{1}{2}\;\left\langle \Omega^{2}\right\rangle {T_{E}}^{2}\right)\exp\left(–R_{2,\text{nano}}T_{E}\right)F_{\text{macro}}\left(T_{E}\right),\;T_{E}\ll t_{c}$$

(2) $$S\approx S_{0}\exp\;\left(-R_{2,\text{micro}}^{*}T_{E}\;\right)\exp\left(–R_{2,\text{nano}}T_{E}\right)F_{\text{macro}}\left(T_{E}\right),\;T_{E}\gg t_{c}$$

where $t_{c}=R_{2,\text{micro}}^{*}/\left\langle \Omega^{2}\right\rangle$ is the characteristic time for the transition between the Gaussian and exponential behaviours, $S_{0}$ is the signal amplitude at $T_{E}=0$, $R_{2,\text{nano}}$ is the transverse relaxation rate due to spin interactions at the molecular/nanoscopic scale, $F_{\text{macro}}\left(T_{E}\right)$ is the effect of macroscopic magnetic field inhomogeneities [38], $\left\langle \Omega^{2}\right\rangle$ is the variance of the field inhomogeneities induced by the magnetic inclusions [17], and $R_{2,\text{micro}}^{*}$ is the transverse relaxation rate induced by the magnetic inclusions at the microscale. A parametric evaluation of these expressions was conducted with a Padé approximation, which is a flexible model‐free signal representation [39] derived from a fraction of two polynomials with coefficients adjusted to satisfy the required asymptotic forms at the short‐time (Gaussian) and long‐time (exponential) limits:

(3) $$S_{\text{Padé}}=S_{0}\exp\left(-\frac{\left\langle \Omega^{2}\right\rangle {T_{E}}^{2}}{2\left(1+\frac{\left\langle \Omega^{2}\right\rangle }{2R_{2,\text{micro}}^{*}}T_{E}\right)}\right)\exp\left(–R_{2,\text{nano}}T_{E}\right)F_{\text{macro}}\left(T_{E}\right)$$

We also used the AW and JC models after parameterization in terms of $\left\langle \Omega^{2}\right\rangle$ and $R_{2,\text{micro}}^{*}$:

(4) $$\begin{array}{c} S_{\mathrm{AW}}=S_{0}\exp\left(-\frac{{R_{2,\text{micro}}^{*}}^{2}}{\left\langle \Omega^{2}\right\rangle }\left(\frac{\left\langle \Omega^{2}\right\rangle }{R_{2,\text{micro}}^{*}}T_{E}+e^{-\frac{\left\langle \Omega^{2}\right\rangle }{R_{2,\text{micro}}^{*}}T_{E}}-1\right)\;\right)\\ \exp\left(–R_{2,\text{nano}}T_{E}\right)F_{\text{macro}}\left(T_{E}\right) \end{array}$$

(5) $$\begin{array}{c} S_{\mathrm{JC}}=S_{0}\exp\left(-\frac{{R_{2,\text{micro}}^{*}}^{2}}{\left\langle \Omega^{2}\right\rangle }\left(\frac{\left\langle \Omega^{2}\right\rangle }{R_{2,\text{micro}}^{*}}T_{E}-\sqrt{1+2\frac{\left\langle \Omega^{2}\right\rangle }{R_{2,\text{micro}}^{*}}T_{E}}+1\right)\right)\\ \exp\left(–R_{2,\text{nano}}T_{E}\right)F_{\text{macro}}\left(T_{E}\right) \end{array}$$

The exponential approximation of the signal is simply

(6) $$S_{\mathrm{Exp}}=S_{0}\cdot \exp\left(-R_{2}^{*}T_{E}\right)\cdot F_{\text{macro}}\left(T_{E}\right)$$

with $R_{2}^{*}=R_{2,\text{micro}}^{*}+R_{2,\text{nano}}$.

### 2.2 Microscopic Underpinnings of Nonexponential Decay

The MRI parameters $R_{2,\text{micro}}^{*}$ and $\left\langle \Omega^{2}\right\rangle$ of the signal decay can be linked to the microscopic properties of the inclusions that contain the magnetic material (e.g., iron‐rich cells), assumed to have a spherical shape. In particular, the mean square frequency deviation $\left\langle \Omega^{2}\right\rangle$ of the magnetic field inhomogeneities generated by randomly distributed inclusions [18, 22, 25] is the following:

(7) $$\left\langle \Omega^{2}\right\rangle =\frac{4}{45}\zeta \cdot \left(1-\zeta \right){\left(\gamma B_{0}\Delta \chi \right)}^{2}\approx \frac{4}{45}\zeta \cdot {\left(\gamma B_{0}\Delta \chi \right)}^{2}$$

where ζ≪1 is the volume fraction of the magnetic inclusions, Δχ is their susceptibility difference with the surrounding tissue (SI units), γ is the gyromagnetic ratio (2.675 × 10^8^ rad/s/T), and $B_{0}$ is the main magnetic field. Note that $\left\langle \Omega^{2}\right\rangle$ is a measure of magnetic field inhomogeneities averaged across an imaging voxel. By contrast, the characteristic Larmor frequency induced by a single sphere is the following: ${\delta \Omega }_{s}=\frac{1}{3}\gamma B_{0}\;\Delta \chi$ [18].

In the framework of the SDR [18] and DNR [22, 25], the relaxation rate is described by the following equations:

(8a) $$R_{2,\text{micro}}^{*}=\lambda_{\mathrm{SDR}}\zeta \gamma B_{0}\Delta \chi ,\;\lambda_{\mathrm{SDR}}=\frac{2\pi }{3\sqrt{3}}\cdot \frac{1}{3}\approx 0.4031$$

(8b) $$R_{2,\text{micro}}^{*}=\lambda_{\mathrm{DNR}}\zeta \gamma^{2}B_{0}^{2}{\Delta \chi }^{2}\tau ,\;\lambda_{\mathrm{DNR}}=\frac{16}{75}\approx 0.2133$$

where $\tau =\frac{r^{2}}{6D}$ is the timescale for water molecules to diffuse away from a spherical magnetic inclusion of radius r. D is the water diffusion coefficient in tissue (1 μm^2^/ms).

In the DNR, the dimensionless parameter $\alpha =\tau \cdot {\delta \Omega }_{s}\propto r^{2}$ [24] represents the amount of spin dephasing induced by the field inhomogeneities over the period 𝜏. In the DNR, the condition α≪1 must be verified. The DNR may therefore apply to distributions of magnetic inclusions with comparatively smaller sizes than the SDR. Note that the relaxation rate of the DNR is parametrically smaller than that of the SDR: $\frac{R_{2,\text{micro},\mathrm{DNR}}^{*}}{R_{2,\text{micro},\mathrm{SDR}}^{*}}=\alpha \frac{\lambda_{\mathrm{DNR}}}{\lambda_{\mathrm{SDR}}}\approx \alpha /2\ll 1$. As a result, the relaxation rate of the MRI data will yield very different properties of the inclusions (Δχ, ζ and τ) under the SDR and DNR.

## 3 Methods

### 3.1 Participant Cohort

MRI data were acquired from five healthy volunteers, including three females (mean age = 36 ± 7 years old). The study received approval from the local ethics committee, and all volunteers gave written informed consent for their participation.

### 3.2 Data Acquisition

MRI data were acquired on a 3‐T whole‐body MRI system (MAGNETOM Prisma; Siemens Medical Systems, Erlangen, Germany) with a 64‐channel receive head coil and a custom‐made multiecho 3D fast low‐angle shot (FLASH) pulse sequence with bipolar readout. To facilitate the detection of a nonexponential signal decay, 16 gradient‐echo images were acquired with a minimal echo time of 1.25 ms and interecho spacing of 1.2 ms. The radio‐frequency (RF) flip angle was 12°, and the repetition time was 23.2 ms. Image resolution was 1.2 mm isotropic. The matrix size of the 3D‐encoded images was 208 × 192 × 144 along the read and two phase‐encode directions. Partial Fourier (Factor 6/8) was used in the phase and partition directions. Data at each *k*‐space location of the 3D images were acquired only once. The multichannel data were combined using Adaptive Combine [40]. Three sets of multiecho gradient‐echo data were acquired on each participant, and the total nominal acquisition time was 18 min 9 s. In addition, data acquisition was also conducted on a water phantom using the same protocol to investigate the effects of system imperfections (e.g., eddy currents) on the transverse signal decay.

For the acquisition of the in vivo data, an optical tracking prospective motion correction system (KinetiCor, Honolulu, HI) was used to minimize image degradation due to head motion [41, 42, 43]. Cardiac pulsation constitutes an additional source of noise in brain relaxometry data, which accounts for up to 35% of the variability of $R_{2}^{*}$ maps across repetitions [44]. To minimize the effect of cardiac‐induced noise, the cardiac pulsation of the participants was recorded using a finger pulse oximeter, and data acquisition was suspended during the systolic period of the cardiac cycle, taken to last for a duration of 300 ms [44]. To preserve the steady state of the magnetization, RF excitation was maintained during the periods of suspension. For a heart rate of 80 beats per minute, this strategy resulted in an increase in scan time by approximately 40%. As a result of these prospective strategies for the correction of head motion and cardiac pulsation, the motion degradation index [43, 45, 46], an index of data quality, lied within a narrow range across participants and did not exceed 3.4 s^−1^ (Figure S1).

Multi‐parameter mapping [47] data were acquired on the same participants to compute maps of the MRI parameter MTsat (magnetization transfer saturation), a semi‐quantitative parameter reflecting tissue myelination with improved contrast between tissue classes, allowing an accurate delineation of subcortical grey matter regions [48]. The protocol comprised three multiecho 3D FLASH scans acquired with magnetization transfer, proton density and *T*_1_ weighting (RF excitation flip angle = 6°, 6° and 21°, respectively; repetition time [T_R_] = 24.5 ms). Eight echo images were acquired for the *T*_1_‐ and proton density‐weighted contrasts and six for the magnetization transfer‐weighted contrast. Image resolution was 1 mm isotropic, and the matrix size was 176 × 240 × 256. B1‐field mapping data were acquired (4‐mm isotropic voxel size, T_R_/*T*_*E*_ = 500/39.1 ms) to correct RF transmit field inhomogeneity effects on the MTsat maps [49, 50]. For correction of image distortions in the B1 map data, B0‐field map data were acquired with a 2D double‐echo FLASH, T_R_ = 1020 ms, *α* = 90°, T_E__1_/T_E2_ = 10/12.46 ms, BW = 260 Hz/pixel and slice thickness = 2 mm. The motion correction system described above was also used here.

### 3.3 Anatomical Imaging Processing

MTsat maps were calculated from the magnetization transfer‐, proton density‐ and *T*_1_‐weighted images with the hMRI toolbox (https://hMRI.info) [51], as described in [47, 52, 53]. MTsat maps were segmented into grey and white matter tissue probability maps using the Statistical Parametric Mapping software (SPM12, Wellcome Centre for Human Neuroimaging, London) [54]. A grey matter mask was computed by selecting voxels with a grey matter probability above 0.95. Globus pallidus (GP), putamen, thalamus and caudate regions of interest (ROIs) were defined from the grey matter mask and the regional labels of the Neuromorphometrics atlas (http://neuromorphometrics.com/). As no label exists for the SN, this region was delineated using an ad hoc procedure from a cuboid placed appropriately in the space of each MTsat map. Within this cuboid, SN voxels were identified from the grey matter voxels labelled as brainstem and ventral diencephalon in the Neuromorphometrics atlas. Beyond subcortical grey matter, the fusiform gyrus was also defined from the grey matter mask and the regional label of the Neuromorphometrics atlas, serving as a reference region with a low concentration of non‐haem iron [5].

### 3.4 Fitting of the Transverse Relaxation Decay

Data were analysed using bespoke analysis scripts written with MATLAB R2021a (MathWorks, Natick, MA). The effect of macroscopic magnetic field inhomogeneities on the gradient‐echo signal ($F_{\text{macro}}$ in Equations 1–6) was corrected with the voxel spread function (VSF) method [38]. The complex four‐dimensional multiecho data from the three repetitions were concatenated along the echo time dimension and denoised using the Marchenko–Pastur PCA (MP‐PCA) method [55, 56, 57], using cubic regions of 5 × 5 × 5 voxels. At each voxel, we removed scaling and additive effects between the signal decays acquired across repetitions due to, for example, head motion in the spatially varying sensitivity profile of the receive coil. To suppress the noise floor in the magnitude images, background voxels outside the head were identified from the segmentation of the first gradient‐echo image using SPM12 [54]. In agreement with the noise behaviour in magnitude data after denoising with the MP‐PCA method [56, 58], the distribution of signal intensities across noise voxels was fitted assuming a Rician distribution, and the resulting value of the noncentrality parameter was deducted from the signal intensities. The signal‐to‐noise ratio (SNR) was calculated as the ratio of the signal intensities in the gradient‐echo images and the square root of the noise variance maps provided by the MP‐PCA algorithm used for image denoising [55, 56, 57].

Fitting of the transverse relaxation decay with the analytical expressions of Section 2.1 was conducted voxel‐wise from the concatenated data, across all echo times, using non‐linear least square minimization with a trust‐region‐reflective algorithm (*lsqnonlin* MATLAB function). $R_{2,\text{micro}}^{*}$ was bounded between 1 and 80 s^−1^ with an initial value of 20 s^−1^ for the signal models of Equations (3)–(5). The $\left\langle \Omega^{2}\right\rangle$ parameter ranged from 100 to 4 × 10^4^ rad^2^/s^2^ for the Padé and AW models and from 100 to 8 × 10^4^ rad^2^/s^2^ for the JC model with an initial value of 10^4^ rad^2^/s^2^ for all of them. $R_{2}^{*}$ from Equation (6) ranged from 0 to 80 s^−1^ with an initial value of 20 s^−1^. $S_{0}$ was bounded between 10 and 2000 with an initial value of 500. $R_{2,\text{nano}}$ was not estimated by the fitting procedure because of the unsuitable range of echo times of the data and was set to 10 s^−1^ instead [22, 32, 33]. As $R_{2,\text{nano}}$ depends on tissue iron concentration, this carries the risk of misattributing the actual value of $R_{2,\text{nano}}$ to $R_{2,\text{micro}}^{*}$.

Although the Padé expression is not strictly speaking a model but a representation of the MRI signal, we henceforth refer to all three analytical expressions (Equations 3–5) as models of the MRI signal for the sake of simplicity. The goodness of fit of each model was estimated from the mean squared error (MSE) of the fit and the Akaike information criterion (AIC), which includes a penalty for model complexity. Lower MSE and AIC values indicate a better model fit. Model parameter estimates for the five ROIs were extracted from all voxels and all subjects after the removal of the voxels with high MSE (>15), indicative of spurious effects in the data such as physiological noise (as reference, the average MSE across all voxels is ~8). We also excluded voxels where the transition from Gaussian to exponential behaviour took place over a timescale $t_{c}<0.5$ ms after RF excitation, too short to be robustly detectable.

### 3.5 Robustness of the Transverse Relaxation Parameter Estimates

We performed a noise propagation analysis to characterize the impact of image noise on the parameter estimates $R_{2,\text{micro}}^{*}$ and $\left\langle \Omega^{2}\right\rangle$. We used the estimates of $R_{2,\text{micro}}^{*}$ and $\left\langle \Omega^{2}\right\rangle$ in the subcortical grey matter voxels of an individual subject to generate synthetic gradient‐echo data according to the Padé signal equation (Equation 3). Three copies of the synthetic multiecho data were generated to mimic the three repetitions of data acquisition conducted in vivo (see Section 3.2). Noise was added to the synthetic data by random sampling of the Rician noise distribution estimated during the fitting of this subject's data. In the in vivo multiecho data, the subcortical voxels were replaced by their corresponding synthetic counterparts. The resulting images underwent the same processing as the in vivo data (see Section 3.4) before estimation of $R_{2,\text{micro}}^{*}$ and $\left\langle \Omega^{2}\right\rangle$ using the Padé model. This procedure was repeated 10 times with different samples of the Rician noise distribution. We calculated the variability (standard deviation) of the $R_{2,\text{micro}}^{*}$ and $\left\langle \Omega^{2}\right\rangle$ estimates across the repetitions and their bias relative to the original estimates obtained from the in vivo data.

To investigate the impact of the value of $R_{2,\text{nano}}$ used in the fitting procedure (Section 3.4), we used the data from one individual subject to estimate $R_{2,\text{micro}}^{*}$ and $\left\langle \Omega^{2}\right\rangle$ with two alternative values $R_{2,\text{nano}}$ within a plausible range (8 and 12 s^−1^ [22, 32, 33]). The estimates of $R_{2,\text{micro}}^{*}$ and $\left\langle \Omega^{2}\right\rangle$ were compared to those obtained with the default value ($R_{2,\text{nano}}$ = 10 s^−1^).

### 3.6 Microscopic Underpinnings of Nonexponential Decay

From the estimates of $R_{2,\text{micro}}^{*}$ and $\left\langle \Omega^{2}\right\rangle$ at each voxel, we attempted to estimate the properties of the magnetic inclusions at the source of the nonexponential decay within brain tissue. This analysis was conducted under the two mutually exclusive scenarios of the SDR and DNR, each providing a different interpretation of the decay curve parameters in terms of microstructural tissue properties.

Under the assumption of the SDR, we estimated the magnetic susceptibility (Δχ) and volume fractions (ζ) of the inclusions (Equations 7 and 8a). Under the assumption of the DNR, where diffusion effects are considered, the model parameters $R_{2,\text{micro}}^{*}$ and $\left\langle \Omega^{2}\right\rangle$ depend not only on Δχ and ζ as in the SDR but also on τ. Because all three properties cannot be estimated separately from $R_{2,\text{micro}}^{*}$ and $\left\langle \Omega^{2}\right\rangle$ alone, only τ was estimated (Equations 7 and 8b).

We conducted non‐parametric Kruskal–Wallis statistical tests of interregional differences in the estimates of Δχ and ζ obtained under the assumption of the SDR (*kruskalwallis* function in MATLAB 2021). Post hoc Tukey's HSD tests were conducted subsequently to identify the pairs of regions at the source of these differences (*multcompare* function in MATLAB). The effect size was computed using Cliff's delta to quantify the magnitude of differences between regions.

### 3.7 Non‐Haem Iron as a Possible Source of the Nonexponential Signal Decay

To investigate if the detected nonexponential decay can be induced by microscopic inclusions of non‐haem iron with cellular sizes, we numerically simulated the gradient‐echo signal decay induced by iron‐rich cells of the SN at 3 T. The numerical simulations were conducted from the cellular distribution of iron in neuromelanin‐pigmented dopaminergic neurons within the volume of an MRI voxel, quantified in 3D with microscopic resolution from a post‐mortem brain [33], and estimates of the magnetic susceptibility of iron and neuromelanin determined using a combination of MRI microscopy and micro X‐ray fluorescence [37].

**FIGURE 1:**
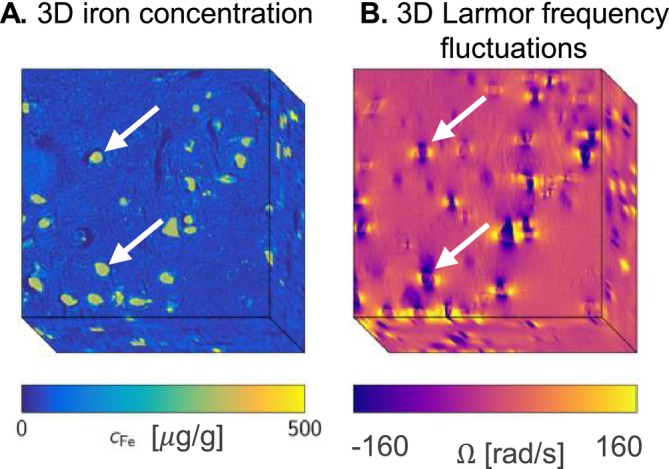
Microscopic iron concentration map obtained from a post‐mortem brain (A, adapted from [33] under the Creative Commons Attribution 4.0 International License), featuring hotspots of iron accumulation inside dopaminergic neurons, as well as diffusely distributed iron outside the neurons. These paramagnetic inclusions lead to local inhomogeneities of the Larmor frequency (B, adapted from [33] under the Creative Commons Attribution 4.0 International License).

We summarize the procedure in detail here. In a first step, the 3D quantitative microscopic iron maps were obtained within the volume of a typical MRI voxel. These maps were generated using histological data from post‐mortem tissue sections of the SN of neurologically healthy human donors. The maps were generated by 3D stacking of semi‐quantitative Perls's stain (10 sections with 10‐μm section thickness), calibrated by quantitative iron measurements of consecutive sections using proton‐induced X‐ray emission microscopy (PIXE). Semi‐quantitative maps were generated from microscopy images using the Beer–Lambert law. Quantitative maps were then derived by calibrating dopaminergic neurons' iron concentrations to PIXE values. A 3D quantitative iron map of the nigrosome N1 was obtained by rigid registration using dopaminergic neuron landmarks and cropped to a dopaminergic neuron‐rich region spanning four voxels of high‐resolution MRI parameter maps (approximately 100 × 440 × 440 μm^3^). These quantitative iron maps show that in the SN, dopaminergic neurons accumulate high levels of iron stored in neuromelanin (Figure 1A). The neuromelanin clusters are about 15 μm in radius and contain approximately 300–1000 μg/g of iron (Figure 1A). In a second step, a susceptibility map was derived from the 3D quantitative iron map by multiplying the iron concentrations in neuromelanin and ferritin by their respective effective susceptibilities (3.3 ppb/(g iron/g tissue) and 1.3 ppb/(g iron/g tissue) [33]). In a third step, the microscopic 3D Larmor frequency inhomogeneities were computed by convolving the susceptibility map with a dipole kernel in Fourier space (eq. 5 in [59]) (Figure 1B).

We then used two methods to simulate the gradient‐echo signal decay generated by this frequency distribution: (1) the SDR approximation and (2) Monte Carlo simulations accounting for water diffusion. Nanoscopic relaxation induced by ferritin and neuromelanin was estimated and taken into account to simulate gradient‐echo decay as described in [27]. The decay resulting from the Monte Carlo simulations was fitted with an exponential for *T*_*E*_ > 10 ms and with Equation ( 3) for all echoes.

The full details of the Monte Carlo simulations are described in [33, 37]. Briefly, the Monte Carlo simulation of the gradient‐echo decay modelled the random, isotropic, unrestricted diffusion motion of water protons in the inhomogeneous field generated by iron‐rich cells in the tissue. The simulation was based on the previously published algorithm of [60], reimplemented in the C++ programming language. It neglected diffusion restriction by cell membranes but used a value of 1 μm^2^/ms for the diffusion coefficient. The simulation included a run of 10^6^ diffusing water protons initiated at random positions within the voxel and was performed with a time step of 0.1 ms and up to a *T*_*E*_ of 50 ms. Phase evolution of each proton was modelled, and the gradient‐echo signal was computed by ensemble averaging. The convergence of the Monte Carlo simulation was verified by comparing the resulting gradient‐echo signal decays simulated by three independent simulations and by comparing the results with a simulation using 10 times more protons.

## 4 Results

### 4.1 Nonexponential Transverse Decay in Subcortical Tissue

Before denoising, the mean SNR of the gradient‐echo data was 47, 59, 63, 46 and 55 in the SN, GP, putamen, thalamus and caudate at minimal *T*_*E*_ (*T*_*E*_ = 1.25 ms) (Figure S2). The SNR of the data at maximal *T*_*E*_ (*T*_*E*_ = 19.25 ms) was 29, 32, 42, 32 and 38, respectively, for the same ROIs.

**FIGURE 2:**
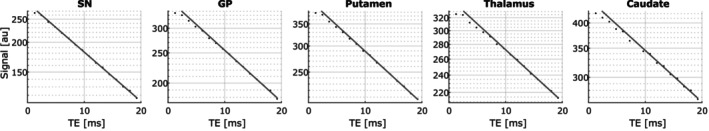
Transverse relaxation decays in one representative voxel of each subcortical grey matter region (semi‐log scale). The line shows the exponential decay fit at long echo times (*T*_*E*_ > 10 ms). At short echo times (*T*_*E*_ ≲ 5 ms), the data deviate from the exponential decay fit, displaying a quadratic decay consistent with the effect of magnetic inclusions on the MRI signal predicted by the theory. Note that with the exception of the exponential fits shown here, this article only shows results from signal models fitted with the data across all echo times.

At short echo times (*T*_*E*_ < 5–10 ms), transverse signal decays in the subcortical grey matter (Figure 2) display systematic deviations from exponential behaviour: The logarithm of the signal exhibits an initial quadratic form, with a transition towards a linear dependence only at longer times. This nonexponential decay is consistent with the effect of magnetic inclusions (e.g., iron‐rich cells, myelin or blood vessels) on the MRI signal predicted by theoretical studies (see [17] for a review). In contrast, the water phantom data do not exhibit evidence of nonexponential transverse signal. This rules out system imperfections (e.g., eddy currents) as the source of the nonexponential behaviour at short echo times in the in vivo data (Figure S3A).

**FIGURE 3:**
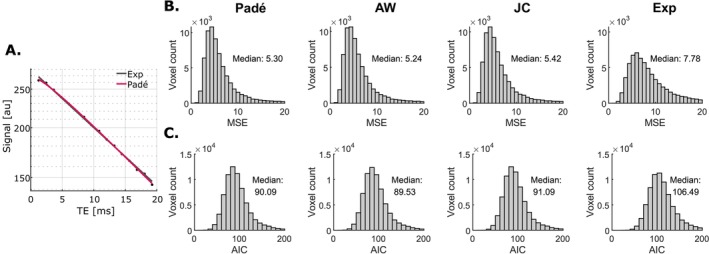
Residual levels across signal models for all subcortical regions analysed. Example fit for a representative voxel in the SN (semi‐log scale). Nonexponential models of transverse relaxation (Padé, AW and JC) can account for the nonexponential decay at short times, leading to an improved fit of the data (A). As a result, the median of residual levels (MSE) is reduced by 30%–35% across subcortical regions, consistently for all nonexponential models (B). This residual decrease leads to a decrease of the median AIC by 15%, beyond that expected from the higher number of parameters of nonexponential models (C).

The nonexponential models of transverse relaxation (Padé, AW and JC) can account for the nonexponential decay of the MRI signal at short times, leading to an improved fit of the data (Figure 3A). The residual levels are largely consistent across the nonexponential models (MSE ~5), 30%–35% smaller than for the exponential fit (MSE ~8) (Figure 3B). Similarly, the AIC decreases by 15% between the exponential (AIC = 106) and nonexponential fits (AIC ~90) (Figure 3C), showing that the residual decrease goes beyond that expected from the higher number of parameters of nonexponential models. In contrast, in the data from the water phantom, the residual levels are 5%–10% smaller with the nonexponential fits (MSE ~46) than with the exponential fit (MSE ~49) (Figure S3B). The average transition time between the Gaussian and exponential behaviours provided by the nonexponential fits is *t*_*c*_ ~0.64 ms, shorter than the minimum echo time of the data. This indicates that signal decay is largely exponential across the range of echo times of the data. The AIC decreases by 1% between the exponential (AIC = 195) and nonexponential fits (AIC ~194) (Figure S3C). The larger MSE and AIC values in the water phantom arise from the higher signal intensities in the GRE images. However, this scaling effect is consistent across all models of the signal decay and cancels out in the estimation of the change in AIC between signal models: these estimates of the change in AIC between the exponential and nonexponential fits are directly comparable with those from the in vivo data.

**FIGURE 4:**
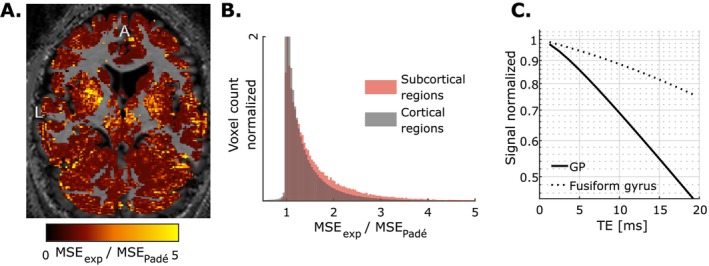
Spatial distribution of the ratio of the MSE obtained from the fits with the exponential and Padé signal models (A). The higher ratio values in subcortical regions (average ~1.6) indicate that stronger deviations from exponential behaviour take place in these areas (B). The stronger nonexponential behaviour in the iron‐rich GP than in the iron‐poor fusiform gyrus is illustrated for a representative voxel (C, semi‐log scale). A—anterior; L—left.

The ratio of the MSE between the exponential and nonexponential fits is higher in subcortical regions (average ~1.6) than in cortical grey matter (average ~1.4, Figure 4A,B), showing that the nonexponential behaviour takes place predominantly there. In particular, this ratio takes a value of 2 in the iron‐rich GP and a value of 1.3 in the iron‐poor fusiform gyrus [5]. Figure 4C shows example signal decays from these two regions.

### 4.2 Estimates of the MRI Signal Model Parameters

**FIGURE 5:**
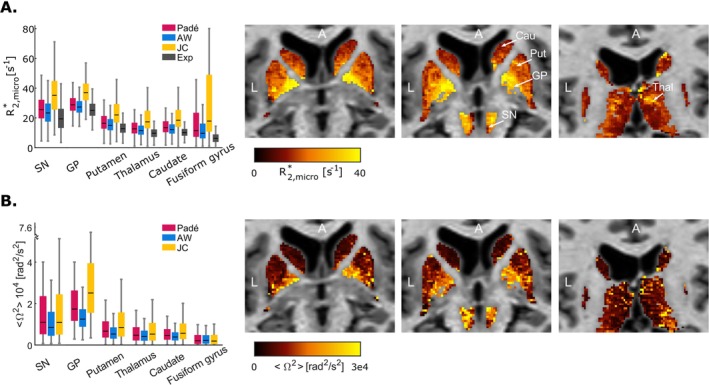
Nonexponential model parameter estimates. Estimates of $R_{2,\text{micro}}^{*}$ (A) and $\left\langle \Omega^{2}\right\rangle$ (B) are highest in the GP, followed by the SN and lowest in the fusiform gyrus, in agreement with histological measures of iron concentration [61] and Equations (7), (8a) and (8b). The example maps of $R_{2,\text{micro}}^{*}$ and $\left\langle \Omega^{2}\right\rangle$ were obtained from the AW model for different slices. A—anterior; Cau—caudate; GP—globus pallidus; L—left; Put—putamen; SN—substantia nigra; Thal—thalamus.

Nonexponential signal decays were reliably detected with *t*_*c*_ > 0.5 ms in 71/81/82/83/77/18% of voxels in the SN/GP/putamen/thalamus/caudate/fusiform gyrus, respectively. In these voxels, the nonexponential models (Padé, AW and JC) lead to estimates of $R_{2,\text{micro}}^{*}$ that are respectively 37%, 30% and 54% higher than the exponential approximation (Figure 5A).

The estimates of $R_{2,\text{micro}}^{*}$ and $\left\langle \Omega^{2}\right\rangle$ are spatially organized and are consistent between anatomical regions (Figure 5). The median values of $R_{2,\text{micro}}^{*}$ obtained with the AW model are 23, 27, 15, 12, 12 and 10 s^−1^ in the SN, GP, putamen, thalamus, caudate and fusiform gyrus. The corresponding values of $\left\langle \Omega^{2}\right\rangle$ are 0.85, 1.25, 0.53, 0.41, 0.38 and 0.22 × 10^4^ rad^2^/s^2^. The higher values in the GP and the SN and lower values in the fusiform gyrus are in agreement with histological measures of iron concentration in brain tissue [61] and with the expected dependence of these parameters on iron content (Equations 7, 8a and 8b). For the AW model, the median transition time between the Gaussian and exponential behaviours is *t*_*c*_ = 2.4, 2.2, 2.7, 2.7, 3.2 and 3.8 ms in these regions.

**FIGURE 6:**
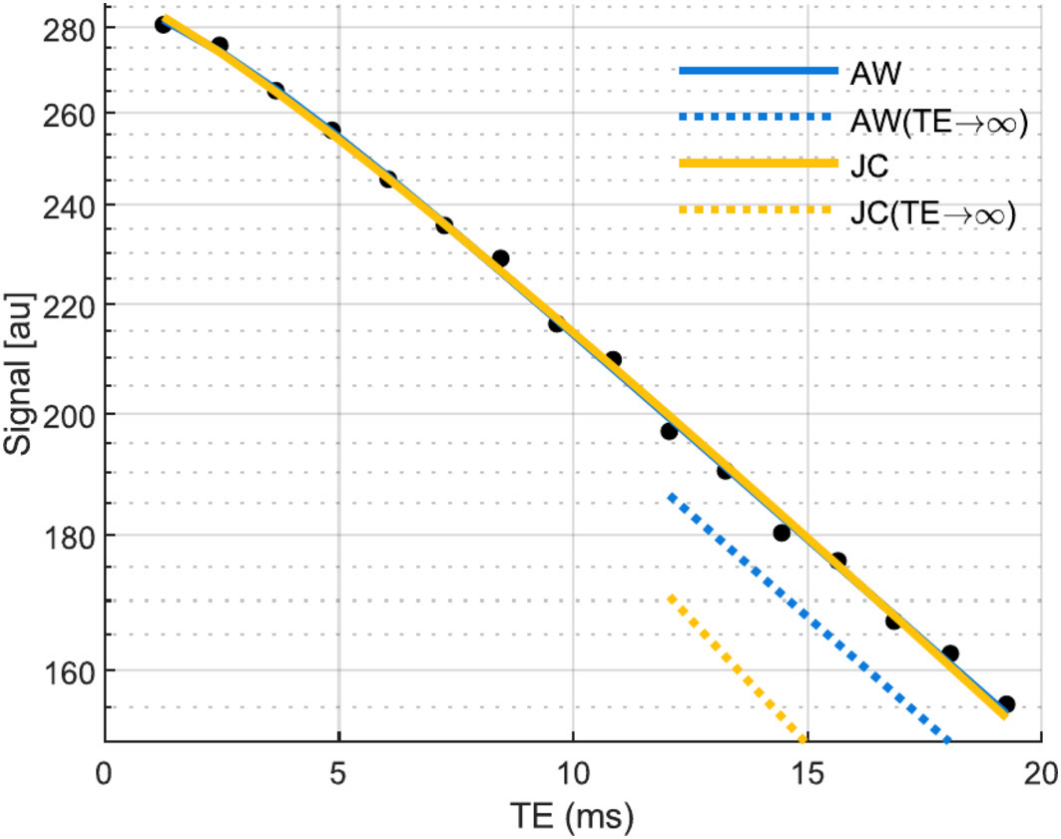
Example transverse relaxation decay from a voxel in the SN and corresponding fits with the AW (blue solid line) and JC (yellow solid line) models (semi‐log scale). For each model, the asymptotic behaviour ($T_{E}\to \infty$) plotted as in [17] (dashed lines) reflects the estimates of $R_{2,\text{micro}}^{*}$. For the AW model, the slope of the experimental data at *T*_*E*_ ~10–20 ms matches that of the asymptotic behaviour. This is not the case for the JC model due to the square‐root term in the JC signal equation: the $R_{2,\text{micro}}^{*}$ estimates provided by the JC model are higher than the decay rate of the data at *T*_*E*_ ~10–20 ms.

The JC model yields systematically higher values of $R_{2,\text{micro}}^{*}$ and $\left\langle \Omega^{2}\right\rangle$ than the AW model and Padé approximation (~31%–73%). This arises from the square‐root term in the JC signal equation (Equation 5), which introduces a broad modulation of the signal over the range of echo times of the data (*T*_*E*_ < 20 ms), that is, a slow transition to a monoexponential decay. As a result, the decay rate of the data at *T*_*E*_ ~10–20 ms differs from the estimates of $R_{2,\text{micro}}^{*}$ at $T_{E}\to \infty$ provided by the JC model: the decay of the data and that of the exponential part of the JC fit have different slopes (Figure 6). On the other hand, the estimates of $R_{2,\text{micro}}^{*}$ from the AW model match the decay rate of the data at *T*_*E*_ ~10–20 ms. Results from the Padé approximation and the AW model are largely consistent.

The results from the noise propagation analysis show that image noise leads to a bias and variability of the $R_{2,\text{micro}}^{*}$ estimates across repetitions below 5% (Figure S4B). For most subcortical grey matter voxels (see histogram in Figure S4A), the bias of the $\left\langle \Omega^{2}\right\rangle$ estimates is within 15%, and their variability is between 15% and 35%. However, the bias and variability of the $\left\langle \Omega^{2}\right\rangle$ estimates can reach 70% and 85%, respectively, in voxels where the transition time between the Gaussian and exponential behaviours ($t_{c}=\frac{R_{2,\text{micro}}^{*}}{\left\langle \Omega^{2}\right\rangle }$) is lower than the minimum echo time of the data.

Setting the value of $R_{2,\text{nano}}$ to 8 s^−1^ in the fitting procedure (instead of the default value $R_{2,\text{nano}}$ = 10 s^−1^) leads to an increase of the $\left\langle \Omega^{2}\right\rangle$ estimates of ~20%–50% (Figure S5B) for the majority of subcortical voxels (0.4 × 10^4^ rad^2^/s^2^ < $\left\langle \Omega^{2}\right\rangle$ < 2 × 10^4^ rad^2^/s^2^ and 5 s^−1^ < $R_{2,\text{micro}}^{*}$ < 25 s^−1^; see Figure S5A). The largest increase in $\left\langle \Omega^{2}\right\rangle$ concerns voxels with small values of $\left\langle \Omega^{2}\right\rangle$ (~0.1 × 10^4^ to 0.2 × 10^4^ rad^2^/s^2^) or $R_{2,\text{micro}}^{*}$ (~1–5 s^−1^). With a value of $R_{2,\text{nano}}$ of 8 s^−1^, the largest increase of the $R_{2,\text{micro}}^{*}$ estimates (50%) concerns voxels with small values of $R_{2,\text{micro}}^{*}$ (~1–5 s^−1^), and the largest decrease (50%) concerns voxels with small values of $\left\langle \Omega^{2}\right\rangle$ (~0.1 × 10^4^ to 0.2 × 10^4^ rad^2^/s^2^) (Figure S5B). For the majority of subcortical voxels, the change in $R_{2,\text{micro}}^{*}$ lies within 10%–20%. Setting the value of $R_{2,\text{nano}}$ to 12 s^−1^ leads to changes of $\left\langle \Omega^{2}\right\rangle$ and $R_{2,\text{micro}}^{*}$ that mirror those with a value of 8 s^−1^ with inverted polarity (i.e., overestimation/underestimation instead of underestimation/overestimation).

### 4.3 Non‐Haem Iron as a Possible Source of the Nonexponential Signal Decay

**FIGURE 7:**
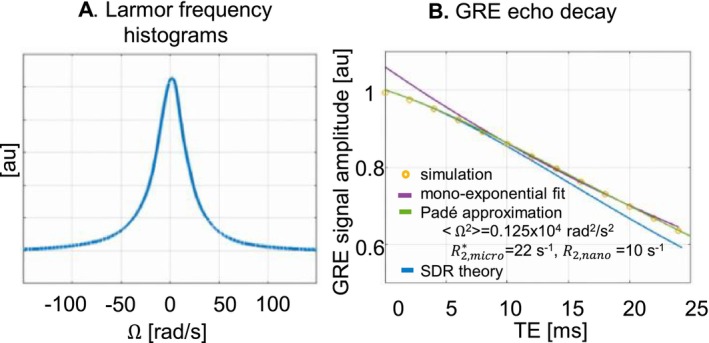
Iron‐rich dopaminergic neurons lead to nonexponential signal decay in the SN at 3 T. As a result of the presence of iron‐rich dopaminergic neurons within the SN (Figure 1), a distribution of ∆Ω ~35 rad/s of the Larmor frequency takes place across the volume of an MRI voxel (A). The resulting Monte Carlo (circles) and SDR (blue line) simulated gradient‐echo (GRE) signal decays (B) deviate from an exponential at short echo times (*T*_*E*_ < 5 ms), consistent with the experimental data (Figure 2). At long echo times (*T*_*E*_ > 10 ms), neglecting water diffusion (SDR) leads to a higher exponential decay rate than when water diffusion is accounted for (Monte Carlo simulation). The Padé approximation (green line) provides a better fit to the Monte Carlo simulated signal ($R_{2,\text{micro}}^{*}$ = 22 s^−1^, $\left\langle \Omega^{2}\right\rangle$ = 0.125 × 10^4^ rad^2^/s^2^) than the exponential model (purple line).

At 3 T, paramagnetic iron‐rich dopaminergic neurons lead to a frequency distribution with a width of ∆Ω ~35 rad/s (Equation 7) across the volume of an MRI voxel (Figure 7A). The gradient‐echo signal decay that results from these field inhomogeneities, computed from Monte Carlo simulations or the SDR, exhibits a deviation from an exponential behaviour at short echo times (*T*_*E*_ < 5 ms; see Figure 7B) consistent with the experimental data (Figure 2). At long echo times, the Monte Carlo simulated signal decay differs from the SDR predictions, suggesting that diffusion effects cannot be ignored for dopaminergic neurons in the SN at 3 T. The Monte Carlo simulated decay is better fitted with the Padé model ($R_{2,\text{micro}}^{*}$ = 22 s^−1^, $\left\langle \Omega^{2}\right\rangle$ = 0.125 × 10^4^ rad^2^/s^2^) than with an exponential, similar to our experimental data (Figures 3 and 5).

### 4.4 Characterization of Magnetic Inclusions Within Subcortical Tissue

From the estimates of the MRI signal parameters $R_{2,\text{micro}}^{*}$ and $\left\langle \Omega^{2}\right\rangle$, we characterized the properties of the magnetic inclusions present within brain tissue at the source of the nonexponential decay under two mutually exclusive scenarios—the SDR and DNR.

#### 4.4.1 Scenario 1

**FIGURE 8:**
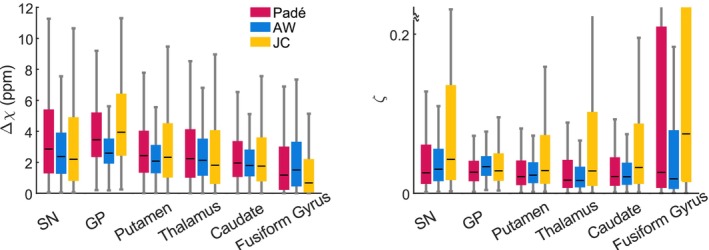
Magnetic susceptibility and volume fractions of magnetic inclusions within brain tissue, estimated from the values of $R_{2,\text{micro}}^{*}$ and $\left\langle \Omega^{2}\right\rangle$ under the assumption of the SDR. Magnetic susceptibilities are larger in the SN and GP (median Δχ~2.5 ppm) compared to the remaining regions (Δχ ~1.6 ppm). The volume fraction of the inclusions (𝜁) ranges between ~0.016 and 0.043 across subcortical regions.

Under the assumption of the SDR (Figure 8), the estimates of $R_{2,\text{micro}}^{*}$ and $\left\langle \Omega^{2}\right\rangle$ were used to estimate the volume fraction (𝜁) and magnetic susceptibility (Δχ) of the inclusions (Equations 7 and 8a). In subcortical grey matter, the median value of Δχ ranges from 1.8 to 4.0 ppm—largest in the GP and SN (~2.6 and 2.4 ppm respectively for the AW signal model), followed by the putamen and thalamus (Δ*χ* ~2.0 ppm) and caudate (Δ*χ* ~1.8 ppm). The fusiform gyrus of the cerebral cortex yields the lowest values of Δχ (~1.5 ppm). The GP and SN show the largest values of 𝜁 (median: 0.034 and 0.030 from the AW signal model, respectively), followed by the putamen (0.023), caudate (0.021), fusiform gyrus (0.018) and thalamus (0.016). The JC model yields estimates of Δχ that are similar to those of the Padé model and approximately 25% higher than those from the AW model. Additionally, the JC model yields estimates of 𝜁 that are about 54% higher compared to the Padé model and approximately 85% higher compared to the AW model. These differences are due to the systematic differences in the $R_{2,\text{micro}}^{*}$ and $\left\langle \Omega^{2}\right\rangle$ estimates highlighted above.

The Kruskal–Wallis tests revealed statistically significant differences in Δχ between at least two ROIs (*F*(5, 76596) = [1579], *p* < 0.001 for the AW model). The corresponding Tukey's HSD test for multiple comparisons found significant differences between Δχ estimates from all ROIs (*p* < 0.01) with small effect sizes, except between the thalamus and putamen where no significance was found. The fusiform gyrus showed the strongest effect sizes (0.12–0.35) compared to the remaining regions.

Similarly, the Kruskal–Wallis tests revealed statistically significant differences in 𝜁 between at least two ROIs (*F*(5, 76596) = [1744], *p* < 0.001 for the AW model). The corresponding Tukey's HSD test showed significant differences between the 𝜁 estimates from the GP and those from the putamen, thalamus and caudate and between SN and thalamus with the largest effect size (>0.30). Other interregional differences were found to be significant (*p* < 0.01) with small effect sizes (<0.20). A detailed overview of this statistical evaluation can be found in Figure S6.

#### 4.4.2 Scenario 2

**FIGURE 9:**
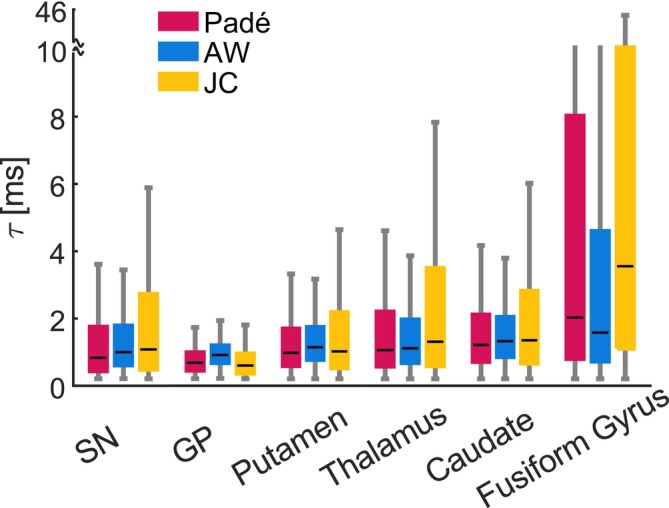
Estimates of the parameter τ, computed from the values of $R_{2,\text{micro}}^{*}$ and $\left\langle \Omega^{2}\right\rangle$ under the assumption of the DNR. The characteristic diffusion times τ are larger in the putamen, thalamus and caudate (median ~1.0 ms) compared to the SN and GP (~0.8 ms). The fusiform shows the largest values of τ (~2.0 ms).

Under the assumption of the DNR, which, for a given $R_{2,\text{micro}}^{*}$ and $\left\langle \Omega^{2}\right\rangle$, requires a greater total amount of iron compared to the SDR, we estimated the parameter τ according to Equations (7) and (8b) (Figure 9). The estimates of τ are larger in the fusiform gyrus (median ~2.0 ms, from the Padé signal model), followed by the putamen, thalamus and caudate (median ~1.0 ms), and finally the SN and GP (median ~0.8 ms). A value of *τ* ~1.0 ms implies a value of ~2.4 μm for the radius of magnetic field inhomogeneities generated by the inclusions. In the fusiform gyrus, this radius is ~3.5 μm (*τ* ~2.0 ms). The JC model yields estimates of τ higher than the Padé model by 26% and than the AW model by 35% because of the systematic differences in the $R_{2,\text{micro}}^{*}$ and $\left\langle \Omega^{2}\right\rangle$ estimates highlighted above.

## 5 Discussion

Here, we provide experimental evidence of nonexponential signal decay due to transverse relaxation in in vivo MRI data from subcortical regions. This signal decay follows a Gaussian behaviour at short echo times with a transition to exponential behaviour at longer echo times. The absence of such decay behaviour in water phantom data rules out system imperfections (e.g., eddy currents) as the source of this effect. Instead, this nonexponential decay is in agreement with theoretical models of the effect of microscopic magnetic inclusions, located within brain tissue, on the MRI signal. These models show an improved fit to the data compared to the widely used exponential model. The strongest deviations from exponential behaviour are found in iron‐rich areas such as the GP and SN. From the values of the model parameters, we estimated the properties of the magnetic inclusions. Numerical simulations of the gradient‐echo signal from post‐mortem maps of iron‐rich dopaminergic neurons in SN show that cells rich in non‐haem iron can be at the source of this decay behaviour. These results illustrate how nonexponential transverse relaxation signal decay can be used to characterize iron‐rich microscopic inclusions from in vivo MRI data.

### 5.1 Nonexponential Transverse Relaxation Decay

Here, we combined a dense sampling of the decay curve with acquisition strategies that mitigate the level of physiological noise in the data [43, 44] to enable the reliable detection of the nonexponential behaviour. Although the mitigation of cardiac‐induced noise led to an increase of scan time by ~40%, alternative strategies are being developed that incur little scan time increase [62, 63].

The lack of evidence for nonexponential signal decay in subcortical regions has been attributed to the short timescale of the transition between the Gaussian and exponential behaviours, below the range of achievable echo times [34]. However, the data presented here show that transverse relaxation decay exhibits a Gaussian behaviour at short echo times (*T*_*E*_ ≲ 5 ms) with a transition towards exponential decay (Figure 2), consistent with theoretical models of the effect on the MRI signal of magnetic inclusions within brain tissue [24, 64]. These models show an improved fit to the data compared with the widely used exponential model (Figure 3). This behaviour was predominantly observed in subcortical grey matter regions (Figure 4), known for their elevated non‐haem iron content. However, nonexponential behaviour was also observed in local areas of cortical grey matter, possibly due to the presence of paramagnetic deoxyhemoglobin in the veins [34] or of non‐haem iron‐rich cells or due to the spurious effect of blood flow on transverse signal decay [65, 66]. Conversely, no deviation from the exponential behaviour was observed in ~20%–30% of subcortical voxels. This may be because of insufficient SNR or because the transition time between the Gaussian and exponential behaviours ($t_{c}$) was too far below the range of echo times of the data.

The highest values of $R_{2,\text{micro}}^{*}$ and $\left\langle \Omega^{2}\right\rangle$ were observed in GP and SN, the areas with the highest iron content (Figure 5) [5, 61, 67, 68], suggesting a dominant role of non‐haem iron in the nonexponential behaviour. In the caudate and thalamus, the lower values of $\left\langle \Omega^{2}\right\rangle$ indicate reduced magnetic field inhomogeneities at the microscopic scale and reflect a broader Gaussian signal decay at short echo times. The characteristic time ($t_{c}$) for the transition to exponential decay was correspondingly longer.

We considered a model‐free Padé approximation and two models of the MRI signal generated by brain tissue with magnetic inclusions. All corresponding expressions fitted the data equally well, with marginal differences between them (Figure 3). Nonetheless, the JC model yields higher estimates of $R_{2,\text{micro}}^{*}$ and $\left\langle \Omega^{2}\right\rangle$ compared to the AW model and Padé approximations. This discrepancy originates from the long transition from the Gaussian to exponential behaviours predicted by the JC model due to the square‐root term in Equation (5). As a result, the estimates of $R_{2,\text{micro}}^{*}$ from the JC model differ from the decay rate of the data at *T*_*E*_ ~10–20 ms (Figure 6).

Here, the effects of the spatial inhomogeneities of the main magnetic field on the gradient‐echo signal decay were corrected using the VSF method [38]. With this method, the correction of the apparent spread of the MRI signal between adjacent voxels with different susceptibilities (e.g., grey/white matter voxels and air/tissue voxels) is based on estimates of the local field gradient between these voxels. The increase of the voxel spread with echo time leads to an apparent signal loss and inaccurate measures of the transverse relaxation rate. The effects of the spatial distortions of the individual echo images are comparably small because the local field gradients (~100 μT/m [69]) are much smaller than the readout imaging gradients (~20–40 mT/m). However, for data acquired using 2D gradient‐echo techniques, the effects of the local field gradients in the through‐plane direction require correction using *z*‐shimming gradients [70].

### 5.2 Characterization of Magnetic Inclusions Within Subcortical Tissue

From the estimates of the two signal model parameters ($R_{2,\text{micro}}^{*}$ and $\left\langle \Omega^{2}\right\rangle$), we attempted to estimate the properties of the magnetic inclusions embedded within brain tissue, which would not have been achievable from the exponential tail of the decay alone. This was conducted under two mutually exclusive scenarios: (1) the SDR (Figure 8) and (2) the DNR (Figure 9), each with distinct analytical expressions to quantitatively link the signal model parameters with the properties of the underlying magnetic inclusions.

#### 5.2.1 Scenario 1

Under the assumption of the SDR, the estimates of the magnetic susceptibility of the inclusions lie in the range Δχ ~1.8–4 ppm across subcortical regions and models of the MRI signal. The largest values of Δχ are encountered in the GP and SN, followed by the putamen, thalamus and caudate in decreasing order (2.6, 2.4, 2.0, 2.0 and 1.8 ppm respectively for the AW model). This ordering follows that of ex vivo measures of bulk iron concentration within the tissue ($C_{\mathrm{GP}}\geq C_{\mathrm{SN}}>C_{\text{Putamen}}>C_{\text{Caudate}}>C_{\text{Thalamus}})$ [5, 61, 71].

The estimates of Δχ derived from the MRI data can be used to determine the iron concentrations within the inclusions themselves and be compared with those obtained from ex vivo studies of non‐haem iron distribution. In the SN, nonexponential relaxation arises mainly from iron bound to neuromelanin in dopaminergic neurons; therefore, $\Delta \chi =\rho \cdot \chi_{{\mathrm{eff}}_{NM}}\cdot {\left[\mathrm{Fe}\right]}_{\mathrm{NM}}$, where ρ ~1.05 g/cm^3^ [72] and $\chi_{eff_{NM}}$ ~2.98 ppm m^3^/kg is the effective magnetic susceptibility of neuromelanin [37]. Using our estimated Δχ ~2.4 ppm (with the AW model), we obtain ${\left[\mathrm{Fe}\right]}_{\mathrm{NM}}$ ~0.77 mg/g, which is consistent with the reported iron concentration of iron in the neuromelanin of dopaminergic neurons (${\left[\mathrm{Fe}\right]}_{\mathrm{NM}}$ ~0.49 mg/g [33, 36, 37]). Because quantitative histological data for other regions are unavailable, we cannot verify the plausibility of our model estimates in those areas. The estimates of Δχ derived from the MRI data differ from the magnetic susceptibility of haem iron in blood capillaries (0.4–0.5 ppm [73, 74]).

The estimates of the volume fraction of the inclusions computed from the MRI data lie in the range ζ ~0.02–0.04 across regions and MRI signal models. In the SN, the volume fraction estimates (ζ ~0.03 with the AW model) are close to those from histological analyses of dopaminergic neurons (0.03–0.12) [33]. However, we highlight that iron‐laden neuromelanin leads to *T*_1_ shortening, an effect that might be at the source of the neuromelanin‐sensitive MRI contrast [75]. In the current study, the RF excitation flip angle (*α* = 12°; see Section 3.2) was chosen as the Ernst angle for a *T*_1_ value of 1000 ms, close to the value of *T*_1_ in the SN at 3 T [76]. It is therefore possible that the volume fraction estimates presented here show a degree of *T*_1_ weighting. The potential dependence of the ζ estimates on the degree of *T*_1_ weighting of the data should therefore be investigated. The MRI‐derived estimates of ζ are also in line with the human capillary vascular volume fraction (~0.02–0.025 [77]).

#### 5.2.2 Scenario 2

Under the assumption of the DNR, we estimated the parameter τ, the decay time of the frequency autocorrelation of water molecules diffusing through the inhomogeneous magnetic field generated by the inclusions. The τ estimates (~1.0 ms) in subcortical grey matter suggest a typical radius r of ~2.4 μm for the magnetic inclusions. This estimate is consistent with the radius of the spherical inclusions reported in other MRI relaxometry studies of excised human grey matter tissue [22]. In particular, the latter study also reported larger values of r in the putamen (3.1 μm), thalamus (3.0 μm) and caudate (2.9 μm), compared to the GP (2.3 μm) as observed here (Figure 9). The MRI‐derived estimate of r is also in the order of a small capillary size (~3.2 μm [78]). However, it is also lower than the typical radius of neuronal or glial cells (5–20 μm in neurons, 5–10 μm in microglia, 2.5–10 μm in astrocytes and 2–5 μm in oligodendrocytes [9, 33, 36, 79]). The separate estimation of Δχ, ζ and τ in the DNR, which can involve estimates of MRI susceptibility in addition to $R_{2,\text{micro}}^{*}$ and $\left\langle \Omega^{2}\right\rangle$, would help verify the validity of the condition $\alpha =\tau \cdot {\delta \Omega }_{s}\ll 1$ required in the DNR. Because the relaxation rate of the DNR is parametrically smaller than that of the SDR $\left(\frac{R_{2,\text{micro},\mathrm{DNR}}^{*}}{R_{2,\text{micro},\mathrm{SDR}}^{*}}\ll 1\right)$, the resulting estimates of Δχ and ζ may differ greatly from those presented here.

Although the SDR may be suitable to describe the dynamics of transverse relaxation in the SN, this may not be the case in other regions. Instead, the most plausible scenario may be that transverse relaxation results from an intermediate dephasing regime between these limiting cases. Therefore, approaches that interpolate between these two limiting cases may allow a more accurate characterization of the magnetic inclusions at the source of nonexponential transverse relaxation in subcortical brain regions [80, 81]. Moreover, brain tissue is inherently complex, involving a distribution of inclusions with varied sizes and susceptibilities originating from different cell types and structures. Some of these inclusions, such as larger cells like neurons, might be better described by the SDR, whereas others, such as smaller cells like glia, might be better described by the DNR. Additionally, the different models of the MRI signal considered here led to systematic differences in the estimates of the magnetic inclusions. Alternative models of the effect of the inclusions on the MRI signal should therefore be considered.

Future quantitative histological studies on cellular iron distributions in different subcortical areas may provide valuable priors for an informed choice of the appropriate model. In combination with the presented acquisition and fitting approach, this could enable the extraction of cellular characteristics non‐invasively from nonexponential MR relaxometry.

### 5.3 Non‐Haem Iron as a Possible Source of the Nonexponential Signal Decay

The strong deviations from the exponential decay in regions with high non‐haem iron content (SN and GP; see Section 5.1) highlight the impact of cells rich in non‐haem iron on the observed nonexponential decay behaviour. In the caudate, significant concentrations of iron have also been reported [5, 61, 67, 68]. Previous studies have suggested that the quadratic behaviour of non‐haem iron may only be detectable at echo times well below 1 ms, below the range of achievable echo times, on the basis that clusters of non‐haem iron are smaller than ~100 nm [34]. As a result, nonexponential signal decay was attributed to haem iron in deoxygenated blood. Here, we conducted numerical simulations of the gradient‐echo signal in the SN, from post‐mortem maps of iron‐rich dopaminergic neurons ~15 μm in size. Like the in vivo data, the simulated transverse signal decay exhibits a transition from a Gaussian to exponential behaviour, on a similar timescale (Figure 7). The similarity of the simulated and in vivo data shows that, in the SN, iron‐rich dopaminergic neurons can lead to nonexponential decay in gradient‐echo data acquired within a conventional range of echo times. The impact of dopaminergic neurons on the decay behaviour in the SN is further highlighted by the similarity of the estimates of intracellular iron concentration obtained from the in vivo data (${\left[\mathrm{Fe}\right]}_{\mathrm{NM}}$ ~0.77 mg/g) and from histological measures of iron concentration in the neuromelanin of dopaminergic neurons (${\left[\mathrm{Fe}\right]}_{\mathrm{NM}}$ ~0.49 mg/g [33, 36, 37]).

In addition to cells rich in non‐haem iron, other magnetic materials undoubtedly also contribute to the nonexponential behaviour. Blood vessels rich in haem iron are a likely contributor due to their paramagnetic properties [20, 30, 34]. However, information on the spatial distribution of cerebral veins within the tissue remains scarce. Myelinated fibres are diamagnetic, and their effects on the gradient‐echo signal have been investigated in theoretical studies [17, 18, 30]. The magnetic properties of myelinated fibres are generally investigated using susceptibility tensor imaging [82, 83, 84]. Because of the anisotropic properties of the susceptibility tensor, the local field inhomogeneities induced by magnetic fibres depend on their orientation relative to the main magnetic field [85, 86]. This orientation dependence has a measurable impact on MRI metrics of, for example, water diffusion [87] or transverse relaxation [88, 89, 90]. In the current work, myelinated fibres may have had a particularly pronounced effect on the thalamus because of its comparatively high myelin content and low iron concentrations [61].

## 6 Conclusions

In this study, we provided experimental evidence of nonexponential transverse relaxation signal decay in in vivo gradient‐echo MRI data from subcortical brain regions at 3 T. The behaviour of the decay is consistent with the effect of magnetic inclusions on the MRI signal predicted by theoretical studies. These theoretical models of the MRI signal yield improved fit with experimental data compared to the widely used exponential model. The larger deviations from exponential decay were observed in iron‐rich subcortical regions (SN and GP). The experimental and numerical results presented here suggest that the observed nonexponential signal decay may originate from cells rich in non‐haem iron such as dopaminergic neurons in the SN. From the estimates of the model parameters, we attempted to characterize the size, volume fraction and magnetic susceptibility of these cells. Nonexponential transverse relaxation signal decay provides new opportunities for the study of iron‐related changes in neurodegenerative diseases non‐invasively from MRI data, with increased specificity.

## Author Contributions

**Rita Oliveira:** conceptualization, methodology, investigation, formal analysis, writing – original draft, writing – review and editing. **Quentin Raynaud:** investigation, writing – review and editing. **Ileana Jelescu:** conceptualization, writing – review and editing. **Valerij G. Kiselev:** conceptualization, writing – review and editing. **Evgeniya Kirilina:** investigation, conceptualization, writing – review and editing. **Antoine Lutti:** conceptualization, methodology, investigation, formal analysis, writing – original draft, writing – review and editing, supervision, project administration, funding acquisition.

## Conflicts of Interest

The authors declare no conflicts of interest.

## Supporting information


**Figure S1.** Distribution of Motion Degradation Index (MDI) across subjects and repetitions.
**Figure S2.** Example maps of the signal‐to‐noise ratio (SNR) of the gradient‐echo data in subcortical grey matter, across the range of echo times of the data.
**Figure S3.** Transverse relaxation decay in a water phantom. Example transverse relaxation decay in a representative voxel (semilog‐scale) (A). The solid line shows the exponential decay fit with the data at long echo times (T_E_ >10 ms) and provides a good fit with the data at short echo times: no signs of non‐exponential decay are apparent. Distribution of the residual levels (MSE) for the different signal models (B). Distribution of the AIC estimates for the different signal models (C).
**Figure S4.** Effect of image noise on the estimates of $R_{2,\text{micro}}^{*}$ and $\left\langle \Omega^{2}\right\rangle$. Histogram of the ($R_{2,\text{micro}}^{*}$, $\left\langle \Omega^{2}\right\rangle$) estimates (A). Bias and variability of the $R_{2,\text{micro}}^{*}$ and $\left\langle \Omega^{2}\right\rangle$ estimates due to image noise, obtained from the noise propagation analysis (B). Bias was computed as the mean deviation between the $R_{2,\text{micro}}^{*}$ and $\left\langle \Omega^{2}\right\rangle$ obtained from the simulated noisy data and the original estimates from the in vivo data. Variability was computed as the standard deviation (Std) of the $R_{2,\text{micro}}^{*}$ and $\left\langle \Omega^{2}\right\rangle$ estimates across the repetitions of the noise simulations. The black lines delineate areas of the plots in the top‐left corner where $t_{c}=\frac{R_{2,\text{micro}}^{*}}{\left\langle \Omega^{2}\right\rangle }<$0.5 ms.
**Figure S5.** Impact of an inaccurate value of the parameter $R_{2,\text{nano}}$ of the fitting procedure on the estimates of $R_{2,\text{micro}}^{*}$ and $\left\langle \Omega^{2}\right\rangle$. Histogram of the ($R_{2,\text{micro}}^{*}$, $\left\langle \Omega^{2}\right\rangle$) estimates with $R_{2,\text{nano}}$ = 10 s^−1^ (default value) (A). Change of the $R_{2,\text{micro}}^{*}$ ($∆R_{2,\text{micro}}^{*}$) and $\left\langle \Omega^{2}\right\rangle$ ($∆\left\langle \Omega^{2}\right\rangle$) estimates with a value of $R_{2,\text{nano}}$ of 8 s^−1^ and 12 s^−1^ (B).
**Figure S6.** Absolute effect size (cliff’s delta) of the differences in Δχ and 34𝜁 between subcortical regions for the AW models. A value of 0 suggests no difference between the two regions, while values closer to 1 indicate stronger associations. The p‐values are not shown given that excluding the pair thalamus and putamen in Δχ, all the remaining pairwise comparisons were significant (due to the large sample size). Note that given that the matrix is symmetric only the upper part is shown.

## Data Availability

The code used for this analysis is publicly available at https://github.com/LREN‐physics/TransverseRelaxation. The data that support the findings of this study are openly available at https://doi.org/10.5281/zenodo.11235235.
