# Supplementary material for: In Vivo Characterization of Magnetic Inclusions in the Subcortex From Nonexponential Transverse Relaxation Decay
Source: NMR Biomed. 2025 May 5;38(6):e70051. doi: 10.1002/nbm.70051 (PMC12053162; doi:10.1002/nbm.70051)
Supplement: Supplementary file 1 — Figure S1. Distribution of Motion Degradation Index (MDI) across subjects and repetitions. Figure S2. Example maps of the signal‐to‐noise ratio (SNR) of the gradient‐echo data in subcortical grey matter, across the range of echo times of the data. Figure S3. Transverse relaxation decay in a water phantom. Example transverse relaxation decay in a representative voxel (semilog‐scale) (A). The solid line shows the exponential decay fit with the data at long echo times (TE >10 ms) and provides a good fit with the data at short echo times: no signs of non‐exponential decay are apparent. Distribution of the residual levels (MSE) for the different signal models (B). Distribution of the AIC estimates for the different signal models (C). Figure S4. Effect of image noise on the estimates of R2,micro* and Ω2. Histogram of the (R2,micro*, Ω2) estimates (A). Bias and variability of the R2,micro* and Ω2 estimates due to image noise, obtained from the noise propagation analysis (B). Bias was computed as the mean deviation between the R2,micro* and Ω2 obtained from the simulated noisy data and the original estimates from the in vivo data. Variability was computed as the standard deviation (Std) of the R2,micro* and Ω2 estimates across the repetitions of the noise simulations. The black lines delineate areas of the plots in the top‐left corner where tc=R2,micro*Ω2<0.5 ms. Figure S5. Impact of an inaccurate value of the parameter R2,nano of the fitting procedure on the estimates of R2,micro* and Ω2. Histogram of the (R2,micro*, Ω2) estimates with R2,nano = 10 s−1 (default value) (A). Change of the R2,micro* (∆R2,micro*) and Ω2 (∆Ω2) estimates with a value of R2,nano of 8 s−1 and 12 s−1 (B). Figure S6. Absolute effect size (cliff’s delta) of the differences in Δχ and 34𝜁 between subcortical regions for the AW models. A value of 0 suggests no difference between the two regions, while values closer to 1 indicate stronger associations. The p‐values are not shown given that excluding the [file NBM-38-e70051-s001.docx]

**Supplementary Material For: “In vivo characterization of magnetic inclusions in the subcortex from non-exponential transverse relaxation decay”**


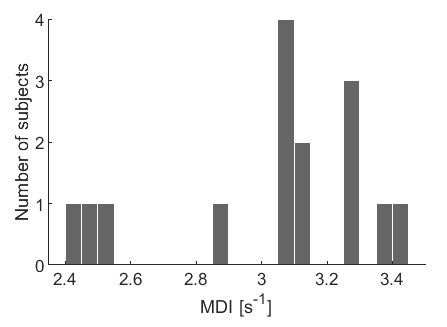


**Figure S1.** Distribution of Motion Degradation Index (MDI) across subjects and repetitions.


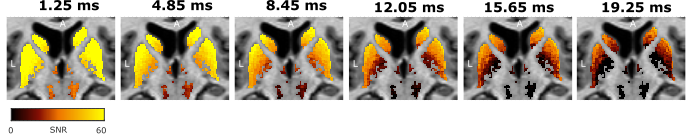


**Figure S2.** Example maps of the signal-to-noise ratio (SNR) of the gradient-echo data in subcortical grey matter, across the range of echo times of the data.


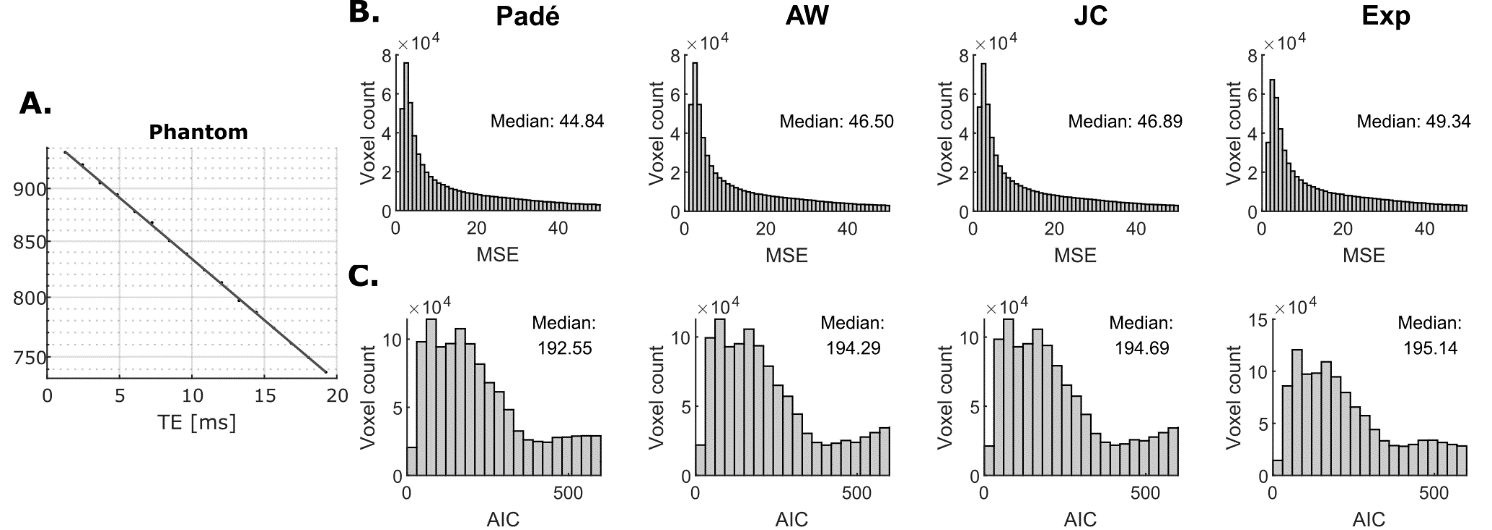


**Figure S3**. Transverse relaxation decay in a water phantom. Example transverse relaxation decay in a representative voxel (semilog-scale) (A). The solid line shows the exponential decay fit with the data at long echo times (T_E_$>$10ms) and provides a good fit with the data at short echo times: no signs of non-exponential decay are apparent. Distribution of the residual levels (MSE) for the different signal models (B). Distribution of the AIC estimates for the different signal models (C).


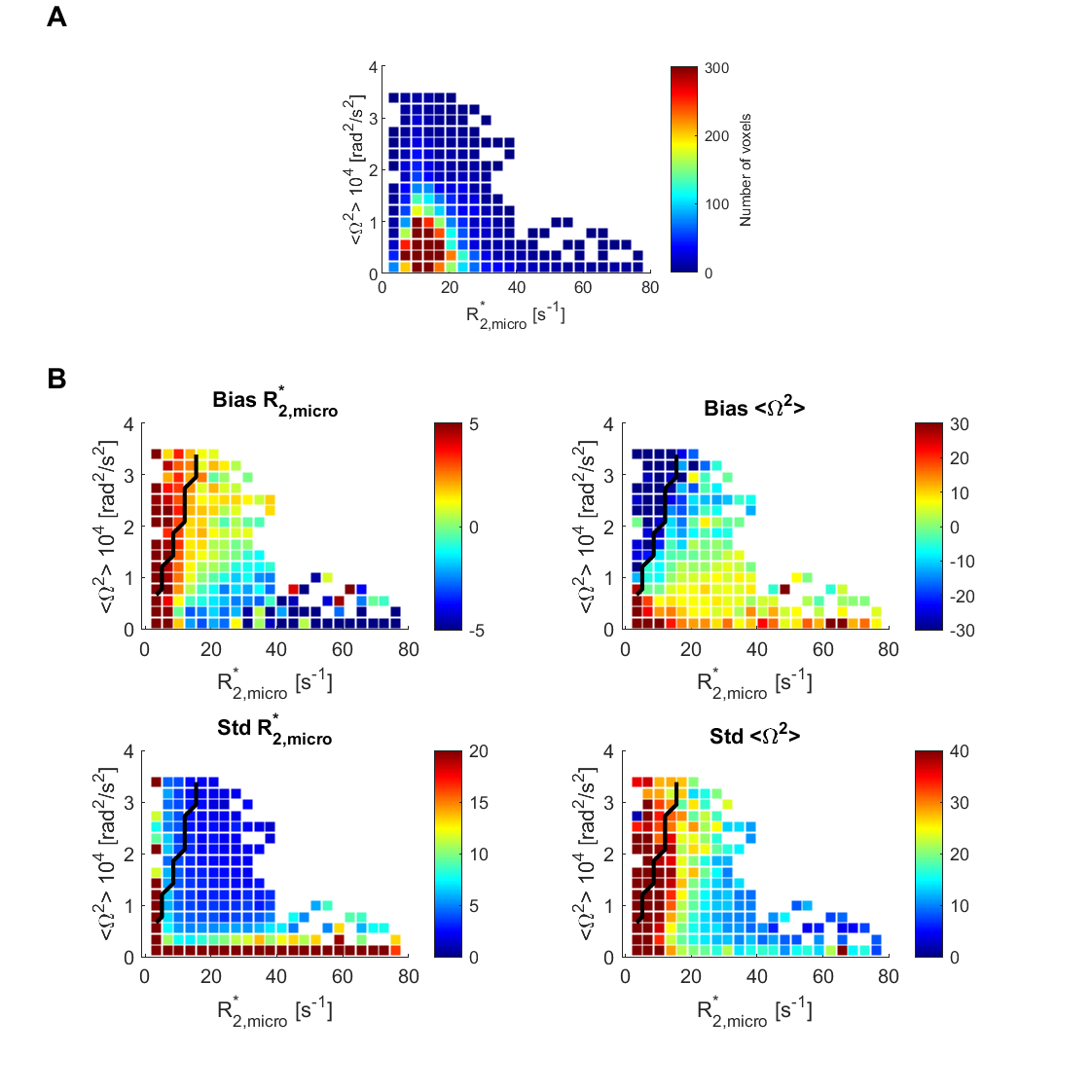


**Figure S4**. Effect of image noise on the estimates of $R_{2,micro}^{*}$ and $\left\langle\Omega^{2} \right\rangle$. Histogram of the ($R_{2,micro}^{*}$, $\left\langle\Omega^{2} \right\rangle$) estimates (A). Bias and variability of the $R_{2,micro}^{*}$ and $\left\langle\Omega^{2} \right\rangle$ estimates due to image noise, obtained from the noise propagation analysis (B). Bias was computed as the mean deviation between the $R_{2,micro}^{*}$ and $\left\langle\Omega^{2} \right\rangle$ obtained from the simulated noisy data and the original estimates from the in vivo data. Variability was computed as the standard deviation (Std) of the $R_{2,micro}^{*}$ and $\left\langle\Omega^{2} \right\rangle$ estimates across the repetitions of the noise simulations. The black lines delineate areas of the plots in the top-left corner where $t_{c}=\frac{R_{2,micro}^{*}}{\left\langle\Omega^{2} \right\rangle}<$0.5 ms.

**
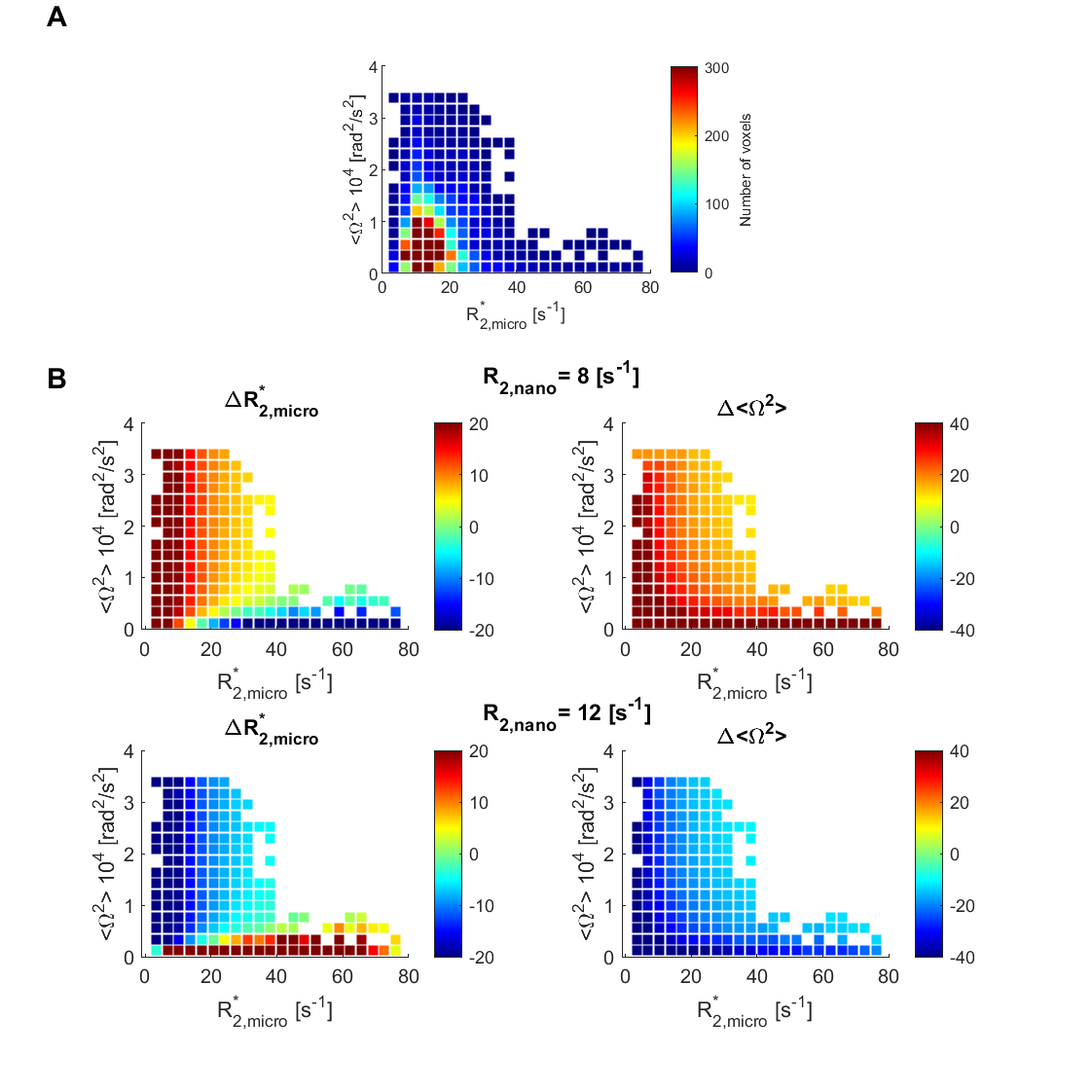
**

**Figure S5.** Impact of an inaccurate value of the parameter $R_{2,nano}$ of the fitting procedure on the estimates of $R_{2,micro}^{*}$ and $\left\langle\Omega^{2} \right\rangle$. Histogram of the ($R_{2,micro}^{*}$, $\left\langle\Omega^{2} \right\rangle$) estimates with $R_{2,nano}$ = 10 s^-1^ (default value) (A). Change of the $R_{2,micro}^{*}$ (${\Delta R}_{2,micro}^{*}$) and $\left\langle\Omega^{2} \right\rangle$ ($\Delta\left\langle\Omega^{2} \right\rangle$) estimates with a value of $R_{2,nano}$ of 8 s^-1^ and 12 s^-1^ (B).


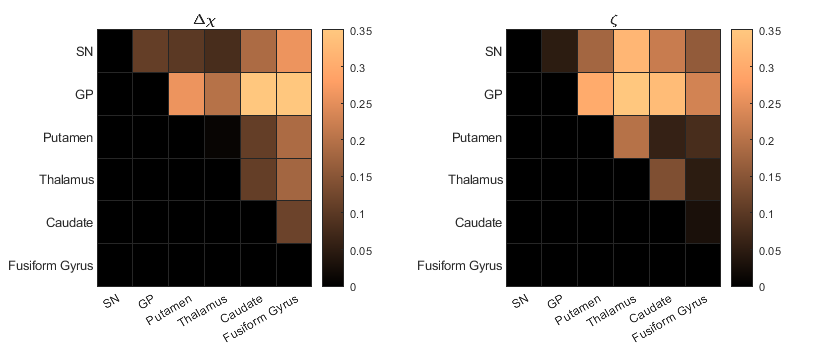


**Figure S6.** Absolute effect size (cliff's delta) of the differences in $\Delta\chi$ and 𝜁 between subcortical regions for the AW models. A value of 0 suggests no difference between the two regions, while values closer to 1 indicate stronger associations. The p-values are not shown given that excluding the pair thalamus and putamen in $\Delta\chi$, all the remaining pairwise comparisons were significant (due to the large sample size). Note that given that the matrix is symmetric only the upper part is shown.
